# KIF2C accelerates the development of non-small cell lung cancer and is suppressed by miR-186-3p via the AKT-GSK3β-β-catenin pathway

**DOI:** 10.1038/s41598-023-30073-5

**Published:** 2023-05-04

**Authors:** Junmei Guo, Wei Zhang, Liping Sun, Hongfang Yu, Yuzhe Wang, Li Feng, Hao Yang

**Affiliations:** 1 Department of Radiation Oncology, Peking University Cancer Hospital (Inner Mongolia Campus) & Affiliated Cancer Hospital of Inner Mongolia Medical University, Huhhot, 010020 China; 2 The Laboratory of Radiation Physics and Biology, Peking University Cancer Hospital (Inner Mongolia Campus) & Affiliated Cancer Hospital of Inner Mongolia Medical University, Huhhot, 010020 China; 3 Abdominal Surgery Department, Peking University Cancer Hospital (Inner Mongolia Campus) & Affiliated Cancer Hospital of Inner Mongolia Medical University, Huhhot, 010020 China

**Keywords:** Cancer, Cell biology, Oncology

## Abstract

This study aimed to explore how kinesin family member 2C (KIF2C) influences the progression of non-small cell lung cancer (NSCLC). The levels of KIF2C and microRNA-186-3p (miR-186-3p) were examined by quantitative real-time polymerase chain reaction (qRT-PCR). Through the utilization of cell counting kit-8 (CCK-8) assay, colony formation assay, wound closure assay, and Transwell assay, NSCLC cell proliferation, migration, and invasion were identified, respectively. NSCLC cell apoptosis was assessed using the TUNEL assay and flow cytometry (FCM) assay. Luciferase reporter analysis was used to investigate the relationship between KIF2C and miR-186-3p. Western blot assays were conducted to investigate the influence of KIF2C on the AKT-GSK3β-β-catenin pathway. The results showed that KIF2C was up‐regulated in NSCLC cells, which predicted poor prognosis. KIF2C overexpression promoted the proliferation, migration, and invasion of NSCLC cells as well as inhibited NSCLC cell apoptosis. KIF2C was as a key target of miR-186-3p. High expression of KIF2C, meanwhile, increased the levels of β-catenin, p-GSK-3β and phosphorylated protein kinase B (p-AKT). KIF2C downregulation and miR-186-3p upregulation reversed these outcomes. As an oncogenic factor, KIF2C is negatively regulated by miR-186-3p and participates in the progression of NSCLC through the AKT-GSK3β-β-catenin pathway.

## Introduction

Around 18% of cancer cases globally are lung cancer. The 5-year survival rate for non-small cell lung cancer (NSCLC), which makes up about 85% of all lung malignancies, is just 19.7%^1,2^.

As a member of the kinesin 13 family member, kinesin 2C (KIF2C) has been demonstrated to be important for cell mitosis and to play a role in spindle assembly, chromosomal aggregation and segregation, and centromere-microtubule junctions. Although KIF2C is substantially expressed in a number of tumor types, its exact mode of action is yet unknown in lung cancer^3^. Study has found that KIF2C participated in the modification of the cytoskeleton during cell invasion and migration, which are important steps in tumor cell proliferation^4^.

Numerous cancers have been shown to have abnormally active Wnt/β-catenin signaling pathways with a wide range of causes. It participates in a range of physiological and pathological processes related to carcinogenesis and embryonic development^5,6^.

Researchers found that miRNA modulated tumor angiogenesis, cell proliferation, differentiation, apoptosis, metastasis, and metabolism, all of which were involved in the incidence and development of tumors^7,8^. Studies have shown that miR-186-3p suppressed the progression of tumors by directly targeting epithelial regulatory proteins in breast cancer cells that were estrogen receptor positive^9^.

The regulatory involvement of miR-186-3p in NSCLC, however, remains unknown.

The aim of this study was to clarify the ability of KIF2C to enhance cancer cell viability, proliferation and invasion in NSCLC by activating the AKT-GSK3β-β-catenin pathway. At the same time, miR-186-3p can negatively regulate the expression of KIF2C, thereby exerting a tumor suppressor effect^10^. Our research revealed the mechanism of KIF2C on NSCLC and the regulatory effect of miR-186-3p on KIF2C, which provides a novel therapeutic target for NSCLC.

## Materials and methods

### Cell culture and transfection

Human immortalized normal lung epithelial cell line BEAS-2B and lung cancer cell lines, including A549, A549-DDP, LLC, PC-9, SHP-77, NCI-H1703 and human embryonic kidney cell line 293T were purchased from the Type Culture Collection of the Chinese Academy of Sciences (Shanghai, China). Cisplatin (DDP) was first synthesized by M. Peyrone in 1845, and later proved to have a killing effect on NSCLC cells. It is now recommended for adjuvant chemotherapy in NSCLC patients after tumor resection. A549 cells are a human adenocarcinoma cell line, while A549-DDP refers to a human adenocarcinoma cell line resistant to DDP. A549-DDP cells were cultured in RPMI-1640 medium (Gibco, BRL, USA). Others were maintained in DMEM medium (Gibco, BRL, USA) at 37 °C in the humidified air with 5% CO_2_.

These media were supplemented with 10% fetal bovine serum (FBS; Invitrogen, CA, USA) and penicillin–streptomycin liquid (100 U/ml penicillin and100 mg/ml streptomycin), and replaced every two to three days. Using 0.25% trypsin (Roche, Basel, Switzerland), cells were passaged once the cell confluence reached 80%. Empty vector (pcDNA-NC), KIF2C overexpression plasmid (PCDNA-KIF2C), small interfering RNA targeting KIF2C (si-KIF2C) and its negative control (si-NC), and miR-186-3p inhibitor and its control (inh-NC), miR-186-3p mimics and their control (miR-NC) were constructed by GenePharma (Shanghai, China). A549 and A549/DDP cells were transfected with these vectors and oligonucleotides using Lipofectamine^®^ 3000 (Invitrogen, CA, USA). Quantitative real-time polymerase chain reaction was carried out after 24 h.

### Quantitative real-time polymerase chain reaction (qRT-PCR)

The total RNA was extracted from these cells utilizing TRIzol reagent (Invitrogen, MA, USA) and complementary DNA (cDNA) synthesis was accomplished using the Prime Script kit (Takara, Beijing, China). The qRT-PCR was performed using SYBR Green (Takara, Beijing, China) in an ABI-7300 RT-PCR system (Applied Biosystems, CA, USA). The ratio to GAPDH was used as an internal control for mRNA, and miRNA expression was normalized utilizing U6. The sequences of the following primers were made:

KIF2C-F: 5′-CAGTGGAATGGGCAGAAGGA-3′

KIF2C-R: 5′-CGGGCAAATTCCCAGTTTGG-3′

miR-186-3p-F: 5′-GCCCAAAGGTGAATTTTTTGGG-3′

miR-186-3p-R: 5′-CAGTGCGTGTCGTGGAGT-3′

GAPDH-F: 5′-GGACTGACCTGCCGTCTAG-3′

GAPDH-R: 5′- TAGCCCAGGATGCCCTTGAG-3′

U6-F: 5′-CTCGCTTCGGCAGCACA-3′

U6-R: 5′-AACGCTTCACGAATTTGCGT-3′

### Cell counting kit-8 (CCK-8) assay

Cells were planted on 96-well plates following transfection at a starting density of 3000 cells per well.

Each well received 10 μL CCK-8 (Dojindo Molecular Technologies, Kumamoto, Japan) at a different monitored time point and incubated with the cells according to the manufacturer's instructions. A multifunctional microplate reader (Tecan Sunrise, Switzerland) was used to detect the absorbance at the wavelength of 450 nm.

### Apoptosis assay

Apoptosis in cells was measured using the Annexin-V-FITC/PI apoptosis detection kit (BD Biosciences, San Jose, CA, USA) in accordance with the manufacturer's instructions. Then the cell suspension was shaken thoroughly for 15 min at room temperature in darkness and supplemented with 10 μL propidium iodide (Sigma Aldrich, St. Louis, USA). Flow cytometry (FCM) was then used to analyze apoptotic cells. Three different runs of each experiment were conducted. A TUNEL assay kit (cat. no. MK1015; Wuhan Boster Biological Technology, Ltd.) was used to detect apoptotic cells according to the manufacturer's instructions. Cells were incubated overnight at 4 °C with anti-neuronal nuclei antibody (cat. no. BM4354; 1:100; Wuhan Boster Biological Technology, Ltd.) according to the manufacturer's instructions. The cells were incubated with TUNEL reaction mixture for 1 h at 37 °C before being washed three times with PBS. Images were captured using an inverted fluorescence microscope at high magnification (400×). Images in three randomly selected areas were quantitated, and the positive cells were measured using Image J software.

### Transwell assay

Transwell migration chambers (8‐μm pore size; Millipore, Billerica, MA, USA) were used to measure cell migration. A total of 2 × 10^5^ cells were suspended in the serum-free DMEM media and planted into the top chamber together. In the lower chamber, DMEM medium with 10% FBS was added. The chambers were incubated for 48 h before removal, and crystal violet was used to stain these cells from the lower chamber following 4% paraformaldehyde for 15 min. Random six fields at high magnification (Olympus, Tokyo, Japan) were chosen, and cell migration was calculated. Additionally, the invasion assays were carried out in Transwell chambers covered with matrigel (Clontech, CA, USA).

### Colony formation assay

At a cell density of 1000 cells/dish, the transfected cells were seeded in triplicate into 60 mm cell culture plates. After 14 days of growth in DMEM medium containing 10% FBS, cells were fixed and stained with 0.1% crystal violet for 30 min. After that, the colonies were meticulously cleaned with PBS until the background was completely clear. The colony formation rate was calculated as follows:

$$ {\text{colony formation rate }}\left( \% \right)\, = \,{\text{amount of colonies}}/{\text{number of seeded cells}}\, \times \,{1}00\% . $$

### Wound healing assay

Transfected cells were planted in six-well plates for 24 h to form a monolayer. After that, each well was scratched with a cross using a sterile pipette tip held vertically. Subsequently, cells were washed with phosphate-buffered saline (PBS) and shaken for 5 min to remove the detached cells. Then the diluted samples and fresh medium were incubated for 72 h. After washing cells with PBS, add the preheated medium or a fresh sample, and take images. A Keyence BZ-9000 microscope (Keyence, Neu-Isenburg, Germany) was used to observe and capture the scratch closure at intervals of 24 h at low magnification.

### Western blot

After cells were collected and fixed, the protein was extracted and examined according to the manufacturer's instructions. Put simply, the lysates were prepared with RIPA lysis buffer (Beyotime Biotechnology, Beijing, China) and centrifuged for 10 min at 4 °C at 12,000 rpm, and the BCA protein assay kit (Pierce, WI, USA) was applied to determine protein concentration in the supernatants. The sample mixed with loading buffer was added to 10% SDS-PAGE and then transferred to a nitrocellulose membrane. The primary antibodies in this part include the Anti-KIF2C antibody, Anti-p-AKT antibody, Anti-AKT antibody, Anti-p-GSK-3β antibody, Anti-GSK-3β antibody, Anti-β-actinin antibody, and Anti-GAPDH antibody. The above antibodies were purchased separately from Cell Signaling Technology (CST, MA, USA). The membranes were washed after being incubated with the primary antibodies overnight and treated for an hour with a secondary antibody labeled with horseradish peroxidase (HRP). The signal was detected with chemiluminescence phototope-HRP kit (Pierce, Rockford, USA USA) after washing the membrane.

### Luciferase reporter assay

In a nutshell, the binding sequences of KIF2C 3'UTR of miR-186-3p, as predicted by the Target Scan Human 7.2 database, were amplified and put into the pmirGLO vector (Promega, WI, USA). The primers were as follows, KIF2C-WT, 5′-GAGGAGTTGTGTTTGTGGAC-3′ (forward), 5′-TGTAAAACGACGGCCAGT-3′ (reverse), and KIF2C-mut (mutated has-miR-186-3p binding site), 5′-GAGGAGTTGTGTTTGTGGAC-3′ (forward), 5′-TGTAAAACGACGGCCAGT-3′ (reverse). A549 cells were co-transfected with the mixture of pluc3′UTR (WT or mut), Renilla luciferase (Promega, Madison, WI) and Lipofectamine 3000 (Invitrogen, CA, USA). After transfection, luciferase activity was measured 48 h later using a dual-luciferase assay system (Promega, WI, USA).

### Statistical analysis

The results were presented as means and standard deviation. Statistics were analyzed using SPSS 20.0 (Chicago, USA). Differences between the groups were compared using the student t-test.

## Results

### NSCLC with a higher expression of KIF2C has a poor outcome

**Figure 1:**
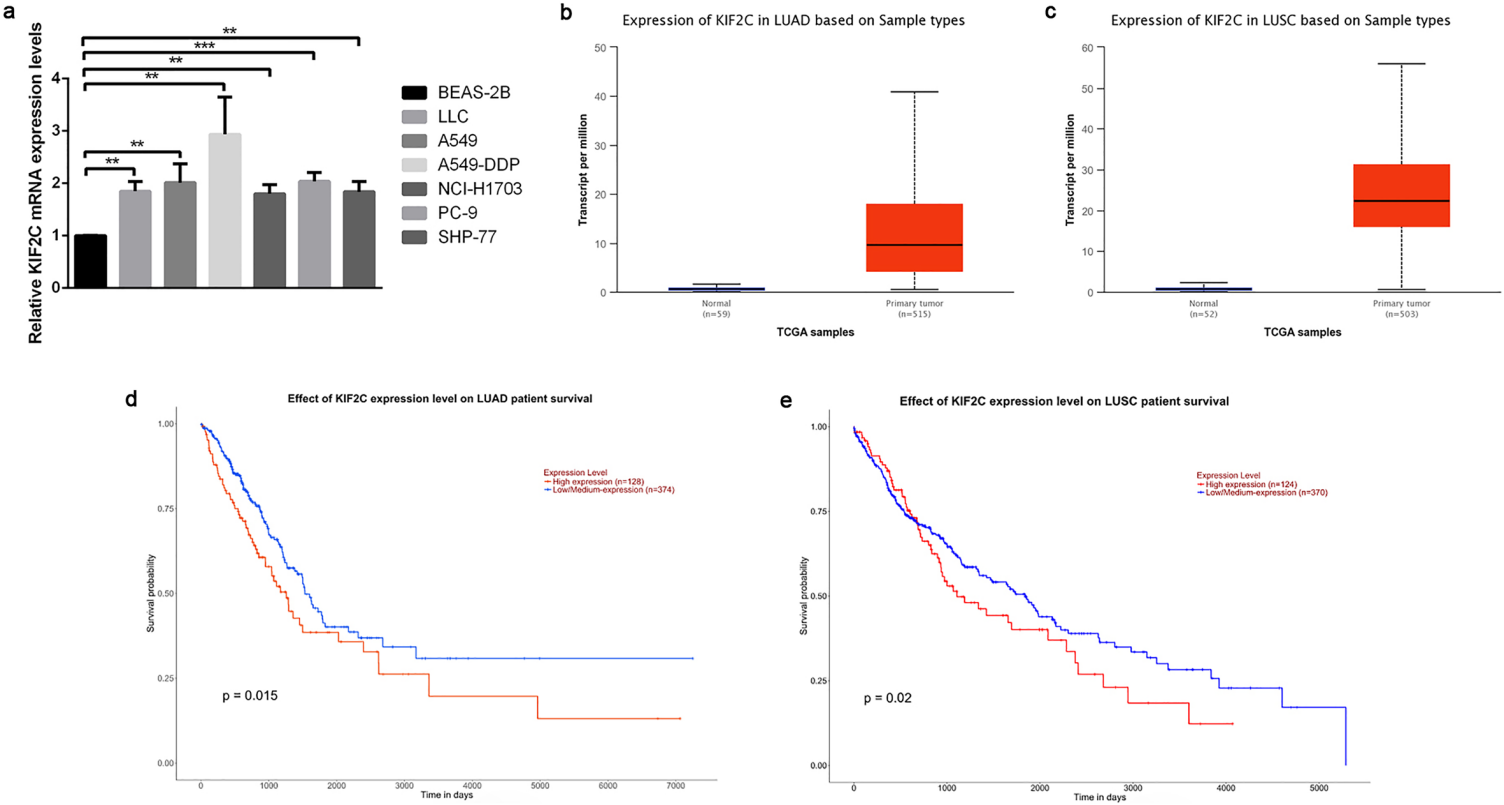
Expression of KIF2C in NSCLC cell lines. (**a**) KIF2C expression is significantly high-expressed in NSCLC cell lines; (**b,c**) KIF2C expression was increased in human lung adenocarcinoma(LUAD) and human lung squamous cell carcinoma(LUSC) tissues; (**d,e**) High KIF2C expression in human lung adenocarcinoma(LUAD) and human lung squamous cell carcinoma(LUSC) tissues were associated with worse prognosis (*P < 0.05, **P < 0.01, and ***P < 0.001).

The purpose of this study was to determine KIF2C expression levels in human lung epithelial cells BEAS-2B and different types of NSCLC cell lines, such as mouse lung adenocarcinoma LLC, human lung adenocarcinoma cells A549, PC-9, SHP-77, A549-DDP, NCI-H1703, through qRT-PCR. In comparison with BEAS-2B, KIF2C expression was significantly higher in NSCLC cells (Fig. 1a). At the same time, KIF2C mRNA levels have been analyzed in human lung adenocarcinomas (LUAD), lung squamous cell carcinomas(LUSC), and normal lung tissue in the Cancer Genome Atlas (TCGA) dataset. According to the results, tumor tissues had significantly higher levels of KIF2C mRNA than normal tissues (Fig. 1b,c). Both high KIF2C expression in LUAD and LUSC tissues were associated with worse prognosis according to the TCGA database (Fig. 1d,e).

**Figure 2:**
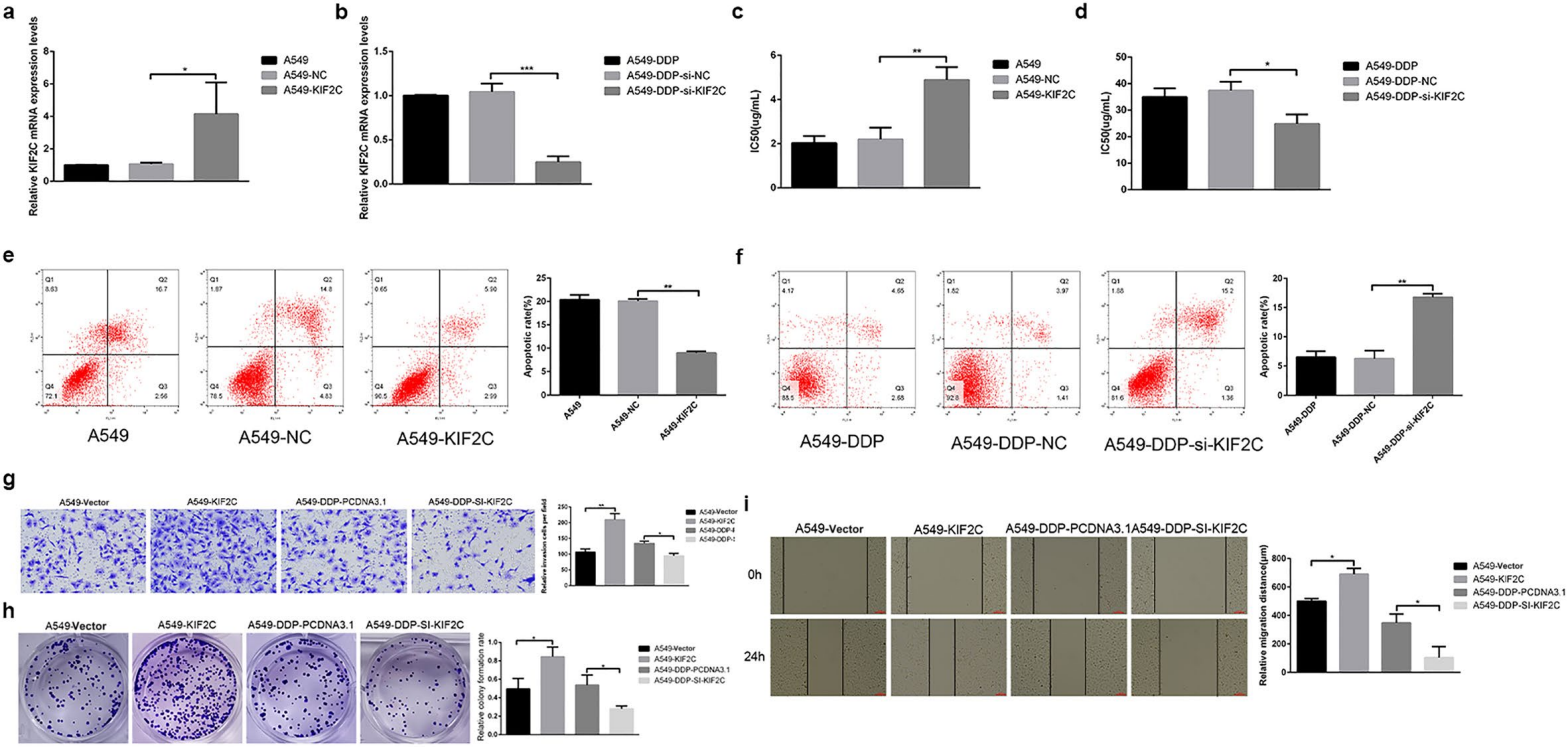
The effects of KIF2C on the proliferation, migration, invasion and apoptosis of NSCLC cells. (**a,b**) Overexpression and silence efficiency of KIF2C evaluated by real-time PCR analysis; (c.d) CCK8 and Colony formation assays were performed to assess the proliferative ability transfected with KIF2C and KIF2C siRNA plasmids; (**e,f**) Flowcytometry assays was used to detect cell apoptosis of overexpression and silence of KIF2C; (**g,h,i**) The effects of KIF2C on migration and invasion were evaluated by the wound healing and transwell assays (*P < 0.05, **P < 0.01, and ***P < 0.001).

KIF2C affects biologically malignant behaviors of NSCLC cellsWith the popularization of computed tomography and other imaging screening methods, adenocarcinoma incidence has increased significantly and has surpassed squamous carcinoma as the most common type. Adenocarcinoma is an invasive carcinoma, which is more likely to relapse and metastasize than squamous carcinoma and difficult to treat^11^. Therefore, we selected LUAD in NSCLC as the research object. Results have showed that A549-DDP cells showed the highest KIF2C expression among NSCLC cells, whereas its corresponding A549 cells showed a relatively low KIF2C expression. As a result, we used plasmid to overexpress KIF2C in low-expressing A549 cells and used small interfering RNA (siRNA) to knock down KIF2C in high-expressing A549-DDP cells. The transfection efficiencies of KIF2C overexpression and silencing were determined by qRT-PCR (Fig. 2a,b). In addition, CCK8 and flow cytometry (FCM) assays were performed to assess the proliferative ability of NSCLC cells transfected with KIF2C and KIF2C siRNA. The results showed that overexpression of KIF2C significantly promoted proliferation and inhibited apoptosis, while silencing of KIF2C inhibited proliferation and promoted apoptosis (Fig. 2c–f). Cancer cell proliferation, migration and invasion are a significant part of cancer progression, so we investigated the effect of KIF2C on these features in NSCLC by performing transwell, colony formation and wound healing assays. We performed cell proliferation, migration, and invasion assays to investigate the biological function of KIF2C in controlling the biologically malignant behaviors of NSCLC cells. The results revealed that, in comparison to the control group, KIF2C overexpression significantly promoted cell proliferation, migration, and invasion, whereas KIF2C knockdown significantly inhibited above cell behavior (Fig. 2g–i).

### KIF2C activated AKT-GSK3β-β-catenin pathway

**Figure 3:**
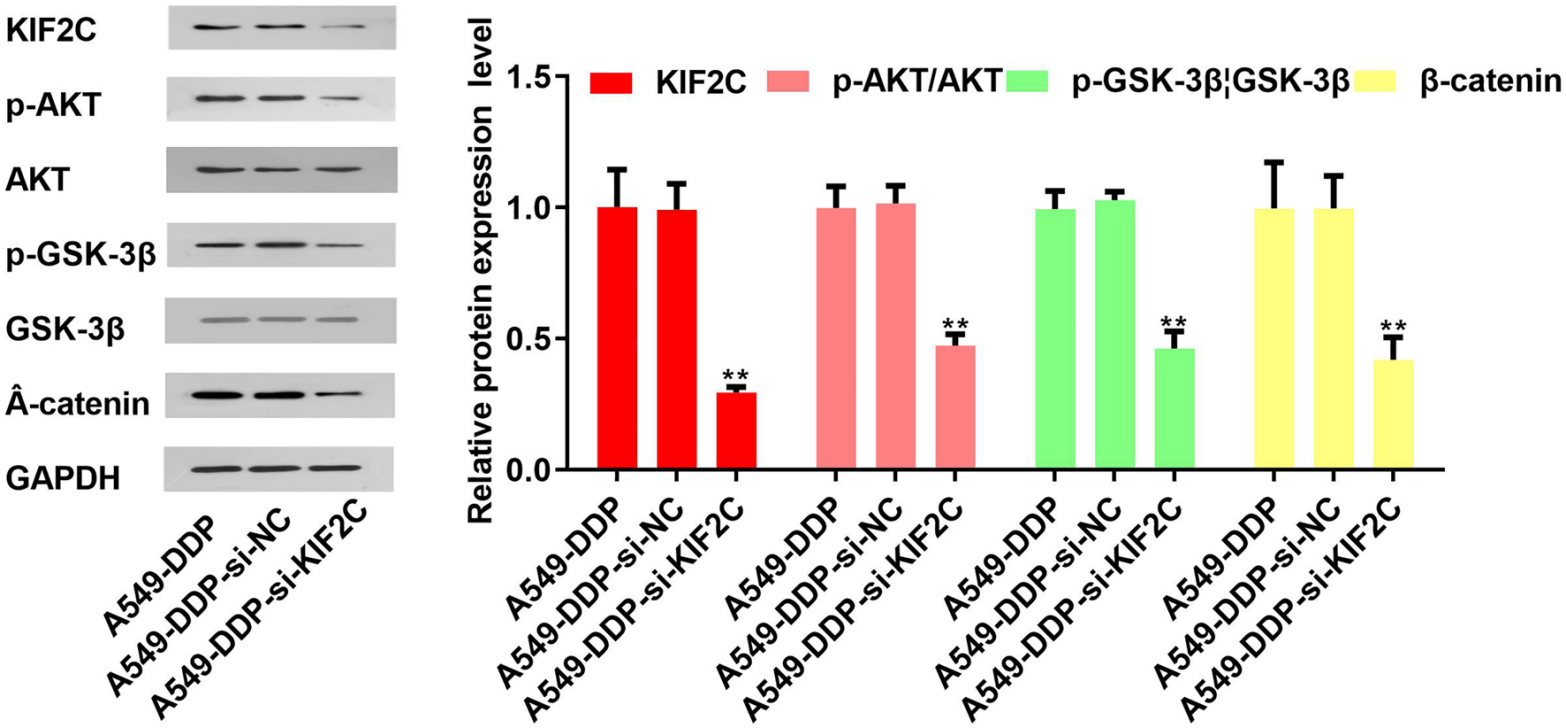
KIF2C was related to the AKT-GSK3β-β-catenin pathway. Western blotting demonstrated that overexpression of KIF2C increased β-catenin, p-GSK-3β and p-AKT level, while KIF2C downregulation had the opposite effects.

Further studies were conducted to investigate how KIF2C influences proliferation, migration, and invasion in NSCLC cells. Wntβ–catenin pathways are broadly involved in promoting tumorigenicity, stemness, and EMT induction^12,13^.Therefore, we investigated if KIF2C could impact the AKT-GSK3β-β-catenin pathway via Western blotting assays. The results demonstrated that overexpression of KIF2C increased β-catenin, p-GSK-3β and p-AKT levels, while the result of KIF2C knockdown was the opposite (Fig. 3).

### miR-186-3p targeted KIF2C regulation

**Figure 4:**
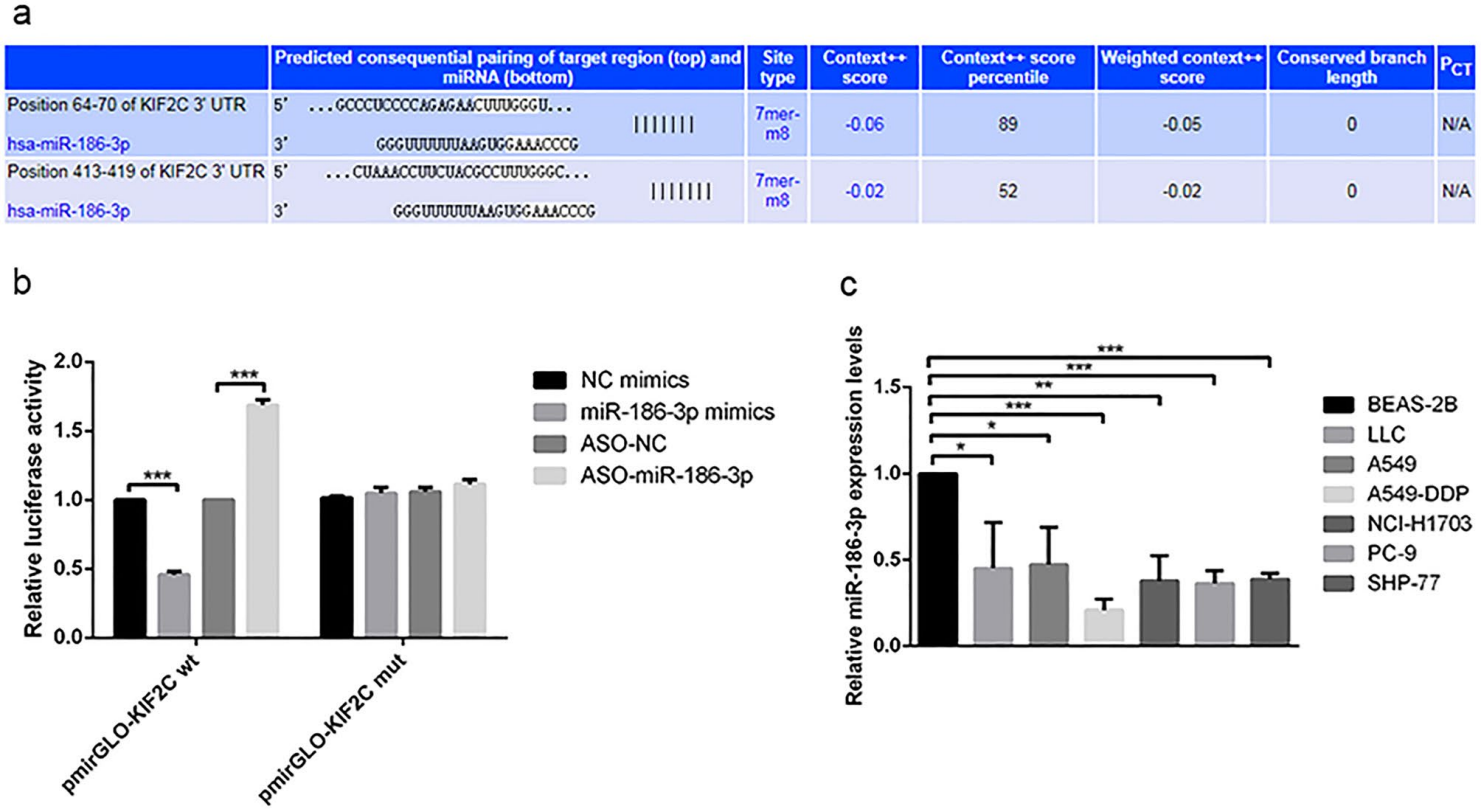
miR-186-3p target KIF2C and expression of miR-186‐3p in NSCLC Cell Lines. (**a**) To further explore the upstream mechanism of KIF2C, the StarBase database (starbase.sysu.edu.cn) was used to predict miRNAs that may regulate KIF2C. It was displayed that there existed binding sites between miR-186-3p and KIF2C 3'UTR; (**b**) subsequently, a dual-luciferase reporter gene assay was conducted for verifying the targeted relationships between KIF2C 3'UTR and miR-186-3p, and it was shown that miR-186-3p overexpression inhibited KIF2C WT's luciferase activity, yet did not significantly affect KIF2C MUT's luciferase activity. (**c**) The expression of miR-186-3p was lower in lung cancer cells.

The StarBase database was chosen to predict the miRNAs that could control KIF2C for a further understanding of the upstream regulatory mechanisms of KIF2C. It was demonstrated that miR-186-3p and KIF2C 3'UTR have binding sites (Fig. 4a, Supplementary file). To verify the targeting relationship between KIF2C 3'UTR and miR-186-3p, we performed a dual luciferase reporter assay. According to the results, overexpression of miR-186-3p reduced the luciferase activity in KIF2C WT cells, but had no discernible effect on KIF2C MUT cells (Fig. 4b). Compared with BEAS-2B cells, miR-186-3p expression was lower in lung cancer cells (Fig. 4c).

### The miR-186-3p/KIF2C axis regulated the biological behaviors in NSCLC cells

**Figure 5:**
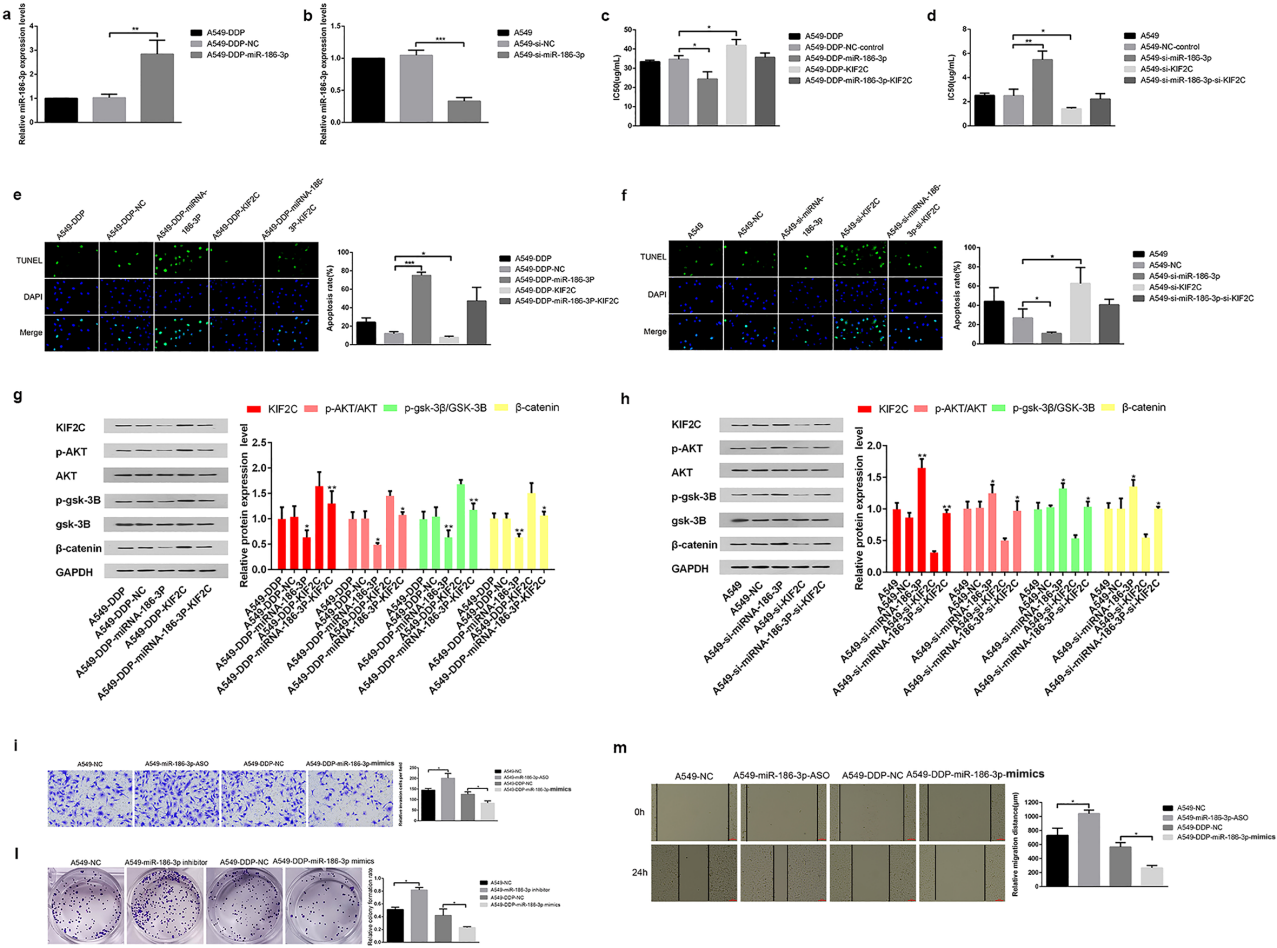
Effects of miR-186-3p/KIF2C axis on NSCLC cell proliferation, migration, and invasion. (**a,b**); The effects of transfection of miR‐186-3p and si- miR‐186-3p on A549 and A549-DDP cell proliferation were determined by qRT-PCR; (**c,d**) the effects of miR‐186-3p silencing and overexpression on A549-DDP and A549 cell proliferation were detected by CCK‐8 assays; (**e,f**) tunel assays were used to detect the cell proliferation and apoptosi; (**g,h**) A549-DDP cells were co-transfected with pcDNA-KIF2C and miR-186-3p mimics, and A549 cells were co-transfected with si-KIF2C and miR-186-3p inhibitors. After transfection, Western blot were used to detect the changes of β-catenin, p-GSK-3β and p-AKT; (**i,l,m**) the effects of KIF2C on migration and invasion were evaluated by the wound healing and transwell assays (*P < 0.05, **P < 0.01, and ***P < 0.001).

An analysis of qRT-PCR results showed that miR-186-3p had been successfully silenced and overexpressed after transfection (Fig. 5a,b). In order to confirm if miR-186-3p contributed to the NSCLC progression by altering KIF2C, A549-DDP cells were co-transfected with pcDNA-KIF2C and miR-186-3p mimics, and A549 cells were co-transfected with si-KIF2C and miR-186-3p inhibitors.

A549-DDP cells were co-transfected with pcDNA-KIF2C and miR-186-3p mimics, whereas A549 cells were co-transfected with si-KIF2C and miR-186-3p inhibitors using CCK8 assays (Fig. 5c,d). To determine whether the proliferation of NSCLC cells was influenced by apoptosis, we performed TUNEL staining.

The results showed that A549-DDP cells transfected with miR-186-3p mimics and A549 cells transfected with si-KIF2C exhibited a higher rate of apoptosis than control cells (Fig. 5e,f). After transfection, western blot was conducted to investigate the effect of miR-186-3p/KIF2C axis on AKT-GSK3β-β-catenin pathway. We found that the levels of β-catenin, p-GSK-3β and p-AKT decreased in A549-DDP cells transfected with miR-186-3p mimics and A549 cells transfected with si-KIF2C compared with those of controls, which indicated that the AKT-GSK3β-β-catenin pathway was inhibited by miR-186-3p/KIF2C axis (Fig. 5g,h). Subsequently, we investigated the effect of miR-186-3p/KIF2C axis on cell proliferation, migration and invasion in NSCLC by performing transwell, colony formation and wound healing assays. The findings revealed that miR-186-3p inhibitors promoted proliferation, migration and invasion of A549 cell compared with the control group, whereas miR-186-3p mimics impeded above features of A549-DDP cells (Fig. 5i,l,m). Overall, these findings suggest that miR-186-3p/KIF2C axis play an important role in biologically malignant behaviors of NSCLC cells.

## Discussion

Incidence and mortality from cancer are most frequently caused by lung cancer worldwide^14^. NSCLC is distinguished by slow cancer cell growth, metastasis, and development^15^. One of the most important symptoms of NSCLC is the metastasis of tumor cells^16^. Therefore, inhibiting tumor cell proliferation and metastasis has a huge auxiliary effect on patients with lung cancer^15^. Researchers found that KIF2C was highly expressed in liver cancer and thyroid cancer, and was a landmark tumor-promoting factor^3,17^. However, the activation mechanism of KIF2C in NSCLC has not been investigated^18^. Therefore, we designed this experiment to explore a new mechanism for restricting the proliferation and metastasis of NSCLC. The study of cancer uses three main approaches: fresh tumor tissue, animal models, and cell cultures. They all have advantages and disadvantages. However, each tumor tissue contains varying amounts of non-malignant cells, and there are restrictions on the acquisition and use of these cells. Animal experiments need to consider species differences. These reasons have made cancer cell lines an important tool for studying lung cancer. Currently an estimated 300–400 human lung cancer cell lines have been established, including small cell (SCLC) and NSCLC. These cell lines have been widely used in the scientific community all over the world and their research has generated more than a thousand citations, and the research results have played a crucial role in revealing the pathogenesis of lung cancer^19^. We found that KIF2C was closely related to chemotherapy resistance of breast cancer through the literature^20^. The latest research has found that KIF2C, as a stemness related genes, may affect the efficacy of chemotherapy and immunotherapy by regulating the tumor microenvironment in LUAD^21^. This also reflects the innovation of our research. In this paper, we presented experimental proof that, in comparison to BEAS-2B, KIF2C was significantly expressed in various kinds of lung cancer cell lines such as LUAD and LUSC. The results of cell experiments showed that KIF2C overexpression significantly enhanced cell proliferation, migration, and invasion while inhibiting cell apoptosis when compared to the control group, whereas KIF2C knockdown prevented cell proliferation, migration and invasion, and enhanced cell apoptosis. The above results illustrated the critical role of KIF2C in the proliferation and metastasis of NSCLC. Meanwhile, according to Western blotting results, overexpression of KIF2C raised the levels of β-catenin, p-GSK-3β, and p-AKT, but downregulation of the protein had the reverse effect. The results demonstrated that KIF2C may contribute to NSCLC growth and metastasis by triggering the AKT-GSK3β-β-catenin signaling pathway.

Many miRNAs act as tumor suppressors or oncogenes in most cancers, and some have tissue-specific expression patterns^8,22^. Researchers found that miR-186 suppressed growth, migration, and invasion of NSCLC^23–25^. MiR-186-3p is involved in a variety of cancer pathogenesis functions^26^. Lu et al. found that miR-186-3p can reduce tamoxifen resistance in breast cancer cells through the EREG axis and can prevent the onset and progression of cervical cancer via targeting IGF1^27^. There are also reports that miR-186-3p can block tumor growth in lung cancer^28^.

**Figure 6:**
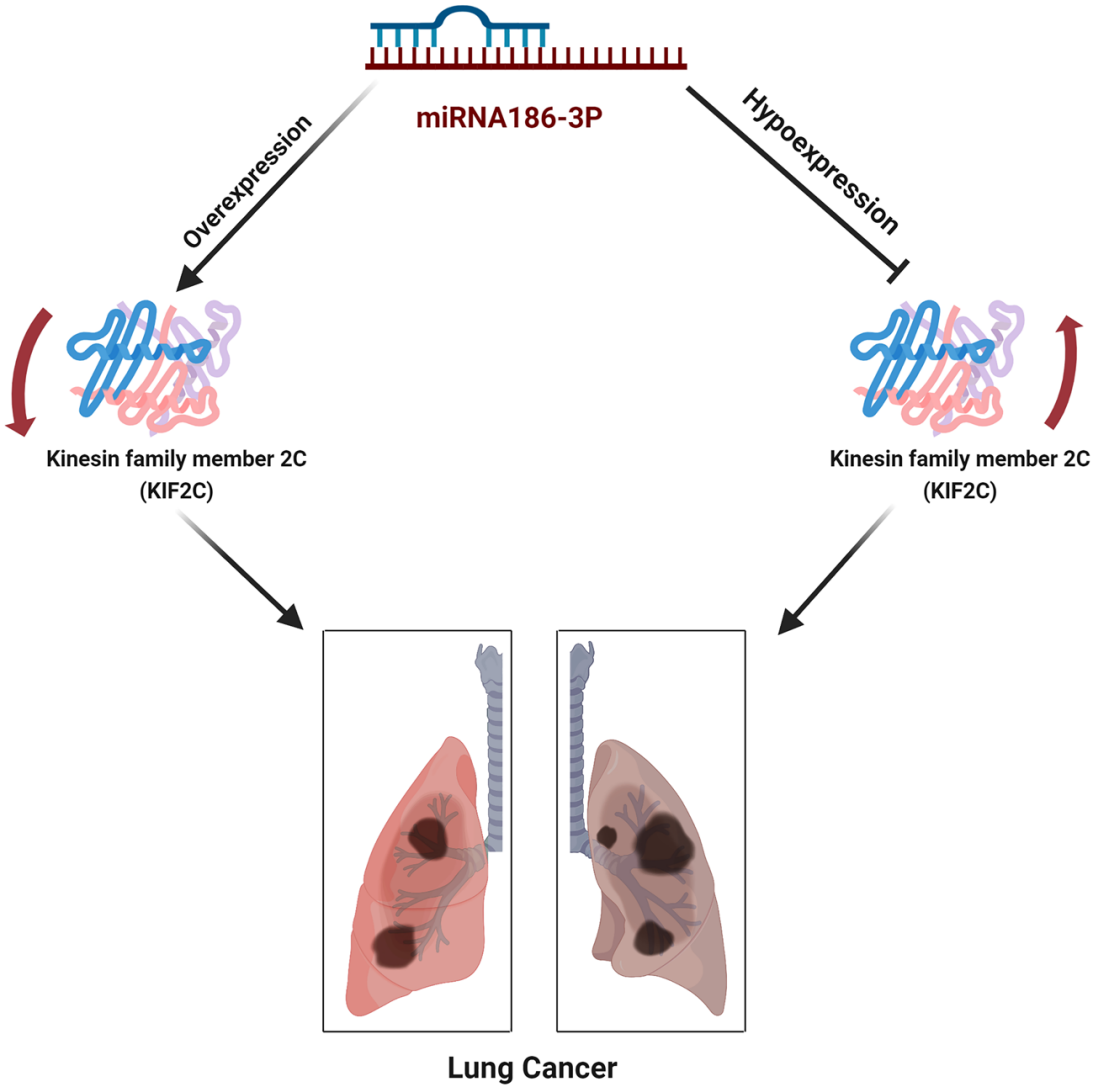
The relationship between KIF2C and miR-186-3p has been shown in the schematic diagram.

We experimentally verified the targeting link between miR-186-3p and KIF2C 3'UTR, and the results suggested that miR-186-3p may control KIF2C expression. In addition to directly inhibiting NSCLC growth and metastasis, miR-186-3p can also reverse the NSCLC-promoting effects of KIF2C. The relationship between KIF2C and miR-186-3p has been shown in the schematic diagram (Fig. 6).

In conclusion, KIF2C is widely expressed in NSCLC, which can promote the proliferation and metastasis of NSCLC. Mir-186-3p can slow down the deterioration of NSCLC by inhibiting the activation of AKT-GSK3β-β-catenin signaling pathway by KIF2C. As a result, we believe KIF2C could be a possible target for NSCLC treatment (Supplementary Information).

## Supplementary Information

Supplementary Table 1.
